# *In situ* oxidation of carbon-encapsulated cobalt nanocapsules creates highly active cobalt oxide catalysts for hydrocarbon combustion

**DOI:** 10.1038/ncomms8181

**Published:** 2015-06-15

**Authors:** Han Wang, Chunlin Chen, Yexin Zhang, Lixia Peng, Song Ma, Teng Yang, Huaihong Guo, Zhidong Zhang, Dang Sheng Su, Jian Zhang

**Affiliations:** 1 Shenyang National Laboratory for Material Science, Institute of Metal Research, Chinese Academy of Sciences, 72 Wenhua Road, Shenyang 110016, China; 2 Institute of New Energy Technology, Ningbo Institute of Materials Technology and Engineering, Chinese Academy of Sciences, 1219 Zhongguan West Road, Ningbo 315201, China; 3 Science and Technology on Surface Physics and Chemistry Laboratory, Mianyang 621907, China

## Abstract

Combustion catalysts have been extensively explored to reduce the emission of hydrocarbons that are capable of triggering photochemical smog and greenhouse effect. Palladium as the most active material is widely applied in exhaust catalytic converter and combustion units, but its high capital cost stimulates the tremendous research on non-noble metal candidates. Here we fabricate highly defective cobalt oxide nanocrystals via a controllable oxidation of carbon-encapsulated cobalt nanoparticles. Strain gradients induced in the nanoconfined carbon shell result in the formation of a large number of active sites featuring a considerable catalytic activity for the combustion of a variety of hydrocarbons (methane, propane and substituted benzenes). For methane combustion, the catalyst displays a unique activity being comparable or even superior to the palladium ones.

## Abstract (web summary)

Palladium is an effective but expensive catalyst used in catalytic converters. Here, the authors show that defective Co_3_O_4_ nanocrystals, synthesized via oxidation of carbon-encapsulated cobalt nanoparticles, display similar or even comparable catalytic activity to palladium for hydrocarbon combustion.

## Graphical abstract

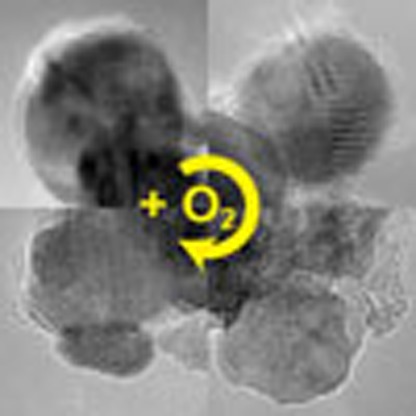

Extensive research has been performed in searching candidates for noble metal catalysts, but there is still a large demand for practically useful and acceptable catalysts for a variety of important reactions. Palladium (Pd) is the most active metal for catalytic combustion of hydrocarbons, while polyvalent metal oxides, especially cobalt oxide (Co_3_O_4_), as the promising candidates still cannot provide a satisfactory activity at the low-temperature region^1^,^2^. Undoubtedly, experimental and theoretical studies have evidenced that the geometry effect at the nanoscale can induce the unexpected properties of an active phase. Low coordinated atoms on stepped and kinked surfaces were reported to strongly bind reactants and often display an outstanding activity for a quantity of structure-sensitive reactions^3^,^4^,^5^. As a cheap candidate to replace noble metals for catalytic combustion, Co_3_O_4_ nanoparticles show a pronounced particle size effect on the reaction pathway of cyclohexane conversion^6^. Smaller nanoparticles with more edge and corner sites would lead to more strongly bound oxygen species to mediate the combustion pathway rather than oxidative dehydrogenation one. Co_3_O_4_ nanocrystals were also found to show a strong crystal plane effect for catalytic combustion of methane (CH_4_), that is, the activity order follows {112}>{011}>>{001} (ref. 7). Hence, the selective synthesis of Co_3_O_4_ nanocrystals containing high-index facets is a key to achieve a high reaction rate being comparable to those of noble metals.

The oxidation reaction of nanostructured metals such as cobalt (Co) and iron can generate nanoscale voids inside the nanocrystals being driven by the Kirkendall effect, which is assigned to the difference in diffusion rates between the anion and cation^8^,^9^,^10^. The atomic diffusion of matter is balanced by an opposite flow of vacancies that are capable to condense into pores and result in deformation or void formation. In case this process is confined into a nano-sized core, both the high surface-to-volume ratio of the particle and the absence of defects in the core remarkably enhance the rate of vacancy injection. The arc-discharging method has been applied to synthesize the carbon-encapsulated Co (Co@C) nanoparticles with a thin graphitic layer as the shell. Using the Co@C core–shell nanocapsules as the starting materials, it should be feasible to produce Co_3_O_4_ nanocrystals with a highly defective surface that may display a satisfactory activity for hydrocarbon combustion reaction.

Here we report a systematic study on the formation process and catalytic performance of highly active Co_3_O_4_ nanocrystals for the combustion of CH_4_ and other hydrocarbons. Co_3_O_4_ nanocrystals are produced via an *in situ* oxidation of Co@C core–shell nanocapsules (carbon content=6.8 wt%; ref. 11). Our studies suggest that the strain gradients induced in the nanoconfined graphitic shell are beneficial not only to generate abundant active sites for enhancing catalytic activity but also to prevent the severe aggregation of metal nanoparticles for keeping stability.

## Results

### Fabrication and characterization of active Co_3_O_4_ nanocrystals

**Figure 1:**
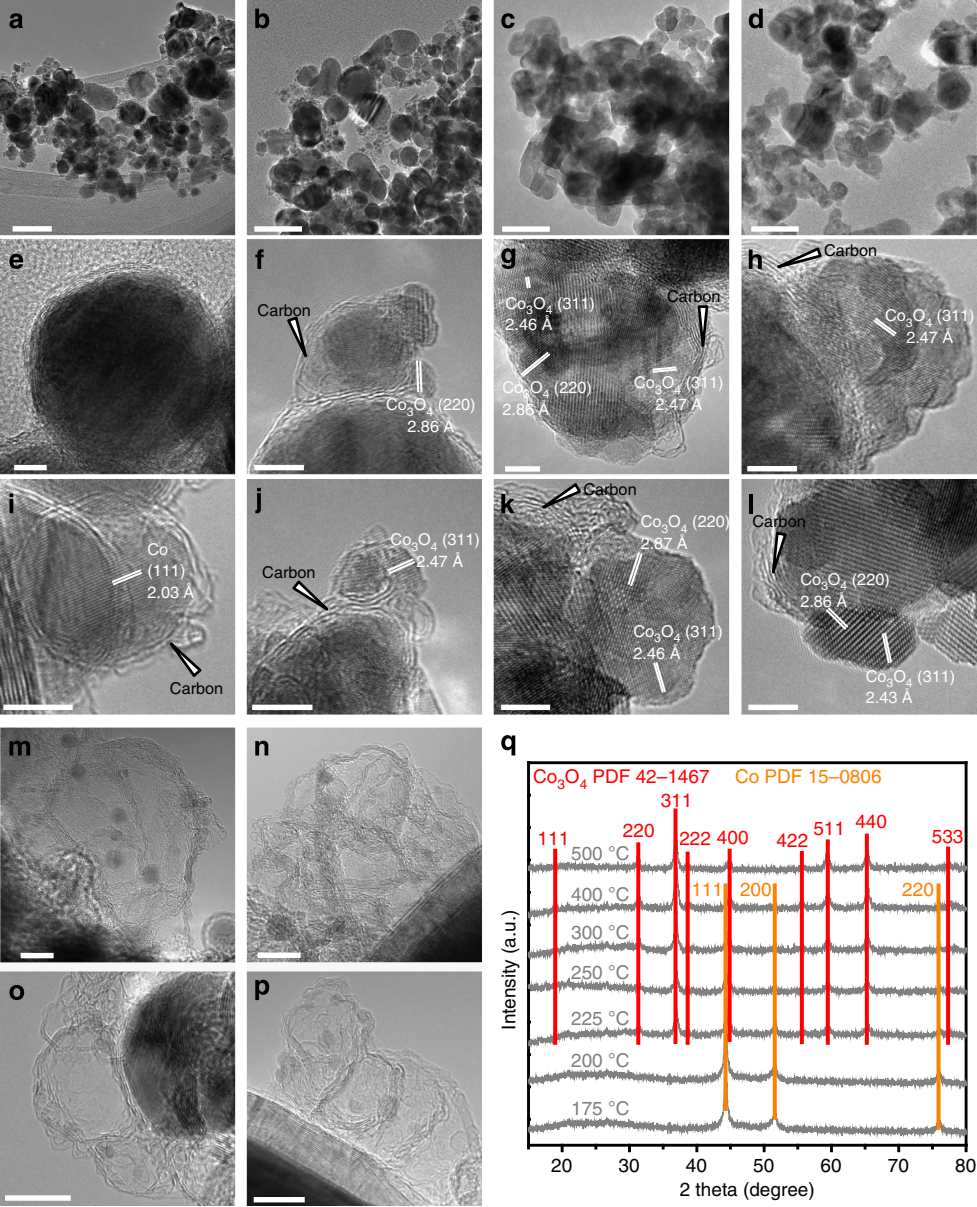
Morphological properties of oxidized Co@C nanocapsules. TEM and HRTEM images of (**a**,**e**,**i**) the pristine and samples that were oxidized at (**b**,**f**,**j**) 200, (**c**,**g**,**k**) 225 and (**d**,**h**,**l**) 250 °C, as well as (**m**–**p**) the residual carbon shells in the oxidized samples at 225 °C after washing by hydrochloric acid. Scale bars, 50 (**a**–**d**), 5 (**e**–**l**) and 10 nm (**m**–**p**). (**q**) XRD spectra of oxidized samples.

To follow the structural change of the Co@C nanocapsules in a flow of diluted oxygen, we used transmission electron microscope (TEM) to detail the morphological properties of the sample after the oxidation at various temperatures. As shown in Fig. 1a–l, the Co@C nanocapsules present as mostly regular spheres with a diameter ranging from 8 to 50 nm. A typical core–shell structure comprises several atomic layers of graphitic carbon as a shell and metallic Co as a core featuring with *d*_002_ spacing of 2.03 Å. After the oxidation at 200 °C, the carbon shell of nanocapsules with a diameter of ∼5 nm was cracked due to the burning by O_2_, while big nanocapsules with highly graphitic shells still kept unchanged. This difference can be related with the chemical reactivity of the carbon atoms on the surface of small nanocapsules with a high surface-to-volume ratio^12^. One metallic core was simultaneously oxidized and disintegrated into several Co_3_O_4_ nanocrystals with lattice distances of 2.44 and 2.86 Å, being assigned to {311} and {220} planes, respectively. These oxide particles spilled from the rupture while the carbon shells were severely deformed (Fig. 1m–p), indicating that a large strain force was produced during the oxidative destruction of nanocapsules. As the treatment temperature was elevated, the Co_3_O_4_ nanocrystals started to aggregate but still kept an irregular shape, while the oxidation of Co nanoparticles without carbon shells is apt to produce oxide particles with a regular shape (Supplementary Fig. 1).

The phase transformation of Co@C nanocapsules in the oxidative atmosphere can be related with the carbon shell as the template. It can be clearly seen in the TEM images (Fig. 1a–l) that defective carbon shell encapsulated or supported Co_3_O_4_ nanoparticles as the transition state. We note that our case obviously differs from the oxidation of exposed metallic Co nanoparticles undergoing successive diffusion of atoms and unrestrained release of lattice stress^13^. Rapid exposure of the metallic core to an oxygen-rich atmosphere will first cause the complete oxidation into Co_3_O_4_, being confirmed by the absence of other Co oxides in powder X-ray diffraction (XRD) patterns (Fig. 1q). Meanwhile, the abrupt transformation from the high-density metallic Co (8.79 g cm^−3^) to low-density spinel Co_3_O_4_ (6.06 g cm^−3^) would cause ∼45% of volume increase^14^. Since the oxidation temperature is far below the melting points of Co and its oxides^15^,^16^, the rapid expansion of the confined core is expected to produce an extensive strain force, inducing the rupture or fragmentation of oxides (Fig. 1a–l). The high-resolution TEM (HRTEM) images of the seriously distorted carbon shells in Fig. 1m–p are particularly important to demonstrate the instant release of deformation force from the core. Quite rough shape and irregular morphology of the extruded Co_3_O_4_ nanoparticles indicate the existence of some high-index facets that are capable to provide a superior activity for the combustion reaction^7^, which can be supported by the diffraction of many high-index crystal planes in the XRD pattern of the used catalyst (Supplementary Fig. 2).

### Catalytic activity and kinetic measurement

**Figure 2:**
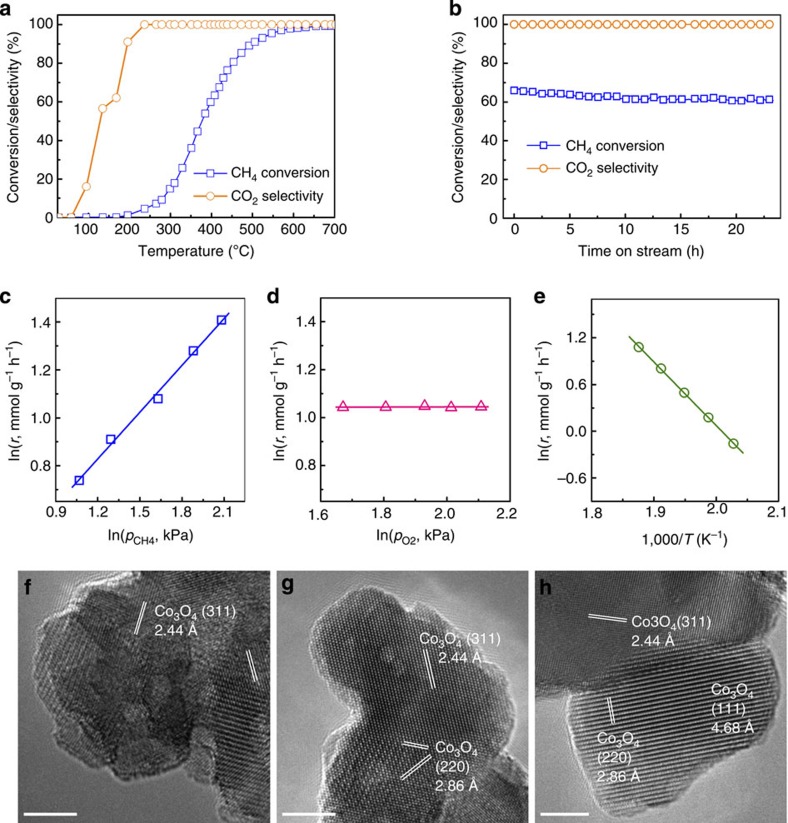
Reaction data. (**a**) Light-off curves of CH_4_ combustion against the increasing temperature and (**b**) catalytic stability at 420 °C under conditions: 0.1 g catalyst, 6.7% CH_4_, O_2_/CH_4_=2.5, helium balance, space velocity 18 l g_cat_^−1^ h^−1^. Dependencies of reaction rate (*r*, mmol g^−1^ h^−1^) on partial pressures of (**c**) CH_4_ (*p*_CH4_) and (**d**) O_2_ (*p*_O2_) at 260 °C, under conditions: 5 mg catalyst, 1.6–5.0 kPa CH_4_, 5.3–8.4 kPa O_2_, helium balance, space velocity 800 l g_cat_^−1^ h^−1^. (**e**) Arrhenius-type plot for CH_4_ combustion under conditions: 5 mg catalyst, 3.0 kPa CH_4_, 7.6 kPa O_2_, helium balance, 220–260 °C, space velocity 800 l g_cat_^−1^ h^−1^. *T*, Kelvin temperature. HRTEM images of the used catalysts after the reaction at temperatures of (**f**) 225, (**g**) 250 and (**h**) 400 °C. Scale bars, 5 nm (**f**–**h**).

Catalytic combustion of CH_4_ (CH_4_+2O_2_→CO_2_+2H_2_O) was used as a probe reaction to evaluate the catalytic properties. The reaction was conducted at 25–700 °C in an oxygen-rich environment with an O_2_ to CH_4_ ratio of 2.5. For each test, the product mixture with CO_2_, CO and residual CH_4_ gives an almost close carbon balance of 100±3%. In a blank experiment without catalyst, the conversion of CH_4_ was negligible even at the temperature as high as 700 °C. Temperature dependency of CH_4_ conversion in the combustion reaction over the catalyst is presented in Fig. 2a, showing that the light-off temperature in the conversion curve was ∼220 °C with 100% selectivity to CO_2_. The temperature at a conversion of 50% (*T*_50_), an important indicator to the activity of a combustion catalyst, is 376 °C, reporting a considerable reaction rate at *T*_50_, that is, 26.8 mmol g^−1^ h^−1^. Such an activity at a low temperature is superior to the transition metal oxides but close to or even higher than the supported Pd catalysts (Supplementary Fig. 3). The time on stream experiment reveals a satisfactory durability at 420 °C. Although a slight decrease in conversion was observed over the initial several hours, the activity had stabilized and remained at above 60% for ∼20 h. During the whole period, the selectivity to CO_2_ did not change and kept at almost 100% (Fig. 2b). The catalyst is also capable to efficiently combust propane, aromatics (benzene, toluene, xylenes, ethylbenzene and styrene) and carbon monoxide^17^ at relatively low temperatures (Supplementary Fig. 4).

To elucidate the reaction mechanism of this process, we conducted the kinetic experiments in a differential reactor to rule out both mass and heat transport effects. Reaction rate approached the same value at a gaseous hourly space velocity of reactants being as high as 18,000 ml g^−1^ h^−1^. The reaction orders of CH_4_ and O_2_ were determined from the logarithm plot of reaction rates versus partial pressure of each reactant, while the apparent activation energy of CH_4_ combustion was derived from the Arrhenius plot (Fig. 2c–e). The reaction orders of CH_4_ and O_2_ were 0.652 and 0.003, respectively, both of which are quite close to those on noble metal catalysts such as Pd, rhodium and so on. (Supplementary Table 1). The tiny reaction order of O_2_ indicates that the reaction rate is independent on the partial pressure of O_2_, and the catalyst surface is saturated by oxygen species without competition between CH_4_ and O_2_ molecules^18^. The apparent activation energy of 68±1 kJ mol^−1^ is even lower than some reported values of activation energies for platinum (67–138 kJ mol^−1^; ref. 19) and Pd catalysts (70–90 kJ mol^−1^; refs 19, 20). This value is also very close to those of some Co_3_O_4_ catalysts^21^,^22^, suggesting an identical structure of the active centres. We therefore attribute the reason for the high catalytic activity to the richness in active sites on the surface of our catalyst. HRTEM images of the used catalysts after reaction at various temperatures were depicted in Fig. 2f–h, indicating that the catalyst surface is highly defective to present a great deal of steps and kinks.

### Structural stability of Co_3_O_4_ catalyst

**Figure 3:**
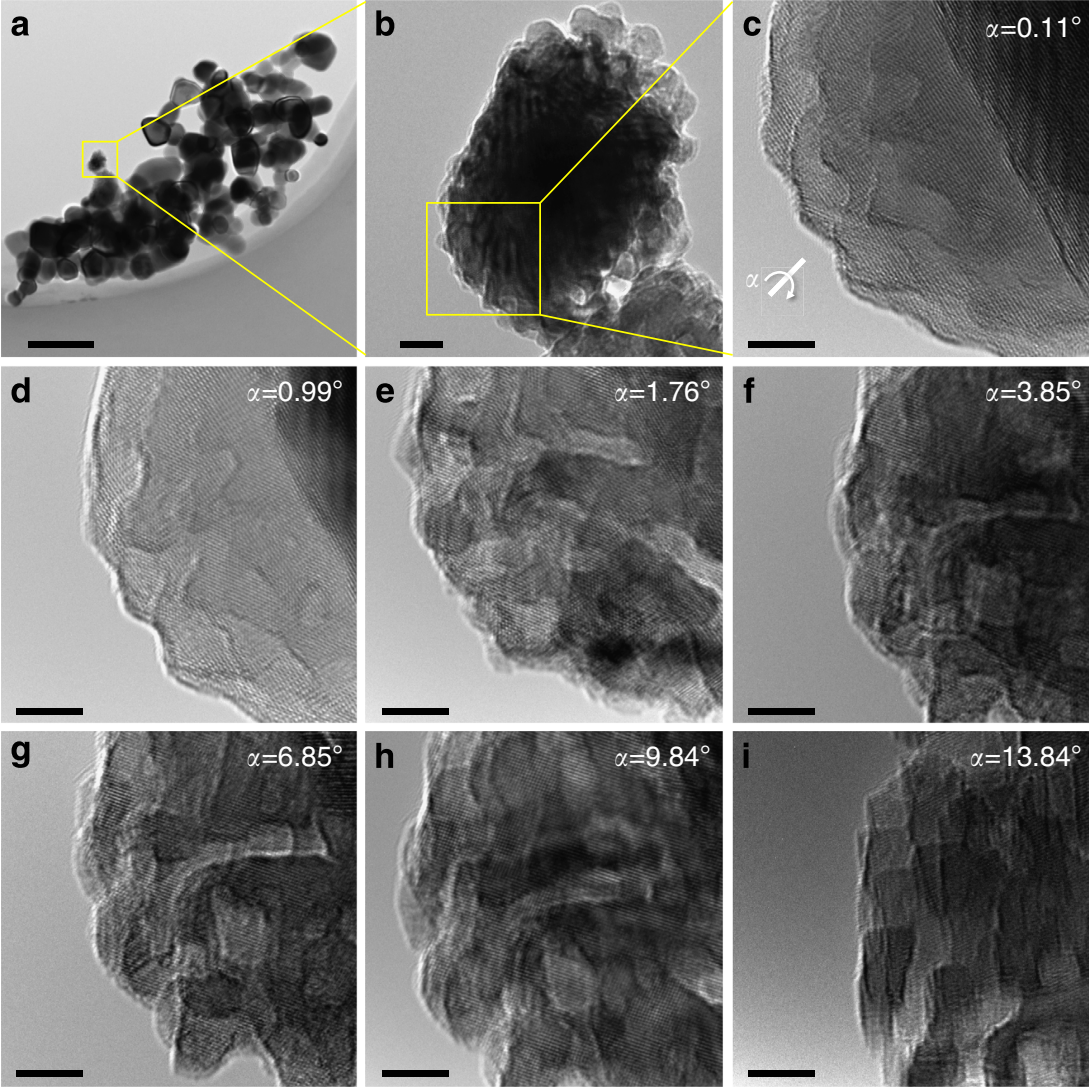
Morphology of catalysts after reaction at 700 °C. TEM images during tilting the sample holder from 0.11° to 13.84°. Scale bars, 200 (**a**), 10 (**b**) and 5 nm (**c**–**i**).

It is worth mentioning that the obtained Co_3_O_4_ nanoparticles displayed an unexpected thermal stability against severe sintering at a high temperature. We detailed the microstructure of the catalyst after the reaction at 700 °C (Fig. 3) by tilting the sample holder inside the TEM chamber. The images clearly reveal the existence of concavities or cracks on the surface and an interconnecting characteristic at a specific angle. The diameter of pores or channels is estimated to be 2–4 nm, being higher than mean free path of reactants and products to allow the efficient mass transport. The nanoscale and devious channels are expected to possess a large number of surface atomic steps on the interior wall that often acted as the catalytically active sites in molecular dissociation and consequential reaction. Although the phase transition at the elevated temperatures led to particle aggregation, the defective carbon shell still plays a pivotal role to prevent serious sintering at the initial stage. The temperature-programmed oxidation test shows that the carbon shells totally disappeared at ∼550 °C (Supplementary Fig. 5). As the metallic Co atoms reacted with O_2_, the oxidation of metallic subsurface at the defective sites was much faster than that in the regions capsulated by well-defined graphitic layers due to the diffusion of oxygen. The atomic diffusion of residual metallic Co into the oxygen-rich phase being driven by the Kirkendall effect finally resulted in a number of disordered voids or channels inside the enlarged particles^9^. Note that the residual carbon shells can also block the approach of reactant molecules to active sites, which would certainly reduce the overall catalytic activity to some extent.

## Discussion

**Figure 4:**
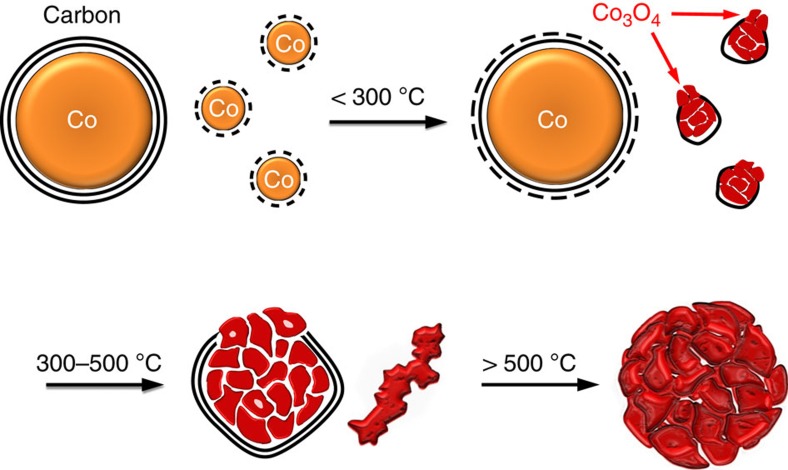
Scheme for the transformation process of the Co_3_O_4_ catalyst. The attenuation of carbon shells in oxygen-containing reaction condition allows the inward diffusion of oxygen and the oxidation of subjacent metallic Co atoms gradually. The volumetric expansion due to phase transformation gives rise to a large strain force to induce the disintegration of bulk oxides. The Co_3_O_4_ nanocrystals were finally formed with nanoscale interconnecting pores at elevated temperatures.

We have demonstrated here the highly active and durable Co_3_O_4_ nanocrystals deriving from Co@C nanocapsules, which efficiently catalysed the combustion of various hydrocarbons especially CH_4_. The formation process of Co_3_O_4_ nanocrystals is illustrated in Fig. 4. The attacks of oxygen result in the partial combustion of carbon shells and consequently the oxidation of subjacent metallic Co atoms, raising Co oxides islands due to the inward diffusion of oxygen. Meanwhile, the immediate volumetric expansion inside the residual carbon shells gives rise to a large strain force to induce the disintegration of bulk oxides. Finally, at elevated temperatures, the oxides nanocrystals with an enlarged particle size and nanoscale interconnecting pores were formed after the complete burning of carbon shells. The defective surface comprising mostly high-index facets endows a considerable activity for combustion of alkanes, and the deviously interconnecting channels allow an efficient mass transfer of reactants. Provided more efforts were made to optimize the structural parameters of nanocapsules, this strategy can be used to synthesize a variety of oxides catalysts for applications in more catalysis processes.

## Methods

### Preparation of Co@C

The Co@C nanocapsules were prepared by evaporating pure Co ingot with the modified arc-discharge technique. The Co ingot with purity 99.99% was used as anode target while a graphite needle with a diameter of 8 mm was served as cathode. As the vacuum of chamber reached 5 × 10^−3^ Pa, the argon and hydrogen with purity of 99.99% were introduced into the chamber to be served as the source of plasma, which lead chamber pressure increase of 0.2 and 0.02 MPa, respectively. Subsequently, 20 ml pure ethanol served as the carbon source was introduced into the chamber. During the arc-discharging process, the Co atoms were evaporated out from the Co ingot to form the Co nanoparticles, and then encapsulated by the carbon atoms decomposed from the ethanol to finally form the Co@C nanocapsules.

### Characterization

The morphology and microstructure characterization was performed by a HRTEM (Techni F20) with a double tilt holder operating at emission voltages of 200 kV. The phase composition of the samples were analysed by powder XRD with Cu-*Kα* (*λ*=0.154,056 nm). The phase transition process of Co@C in oxygen-containing condition was simulated by the temperature-programmed oxidation analysis in air on a thermogravimetric analyser (Netzsch 449F3) at a heating rate of 10 °C min^−1^, which the outlet attached to an AVI Omnistar 200 mass spectrometer to monitor the emergent gases. The adsorption–desorption isotherms of nitrogen were performed on a Micromeritics ASAP 2020 instrument. The CO chemisorption was carried out on a Micromeritics AutoChem II 2920, and the procedure was the same as that described in the reference^23^. The results were shown in Supplementary Fig. 6a,b.

### Catalytic evaluation

The catalytic combustion reaction was carried out in an immobilized bed quartz reactor (8 mm i.d.) in a tri-heating system at the atmospheric pressure. Co@C (100 mg) or carbon-free Co powder diluted with quartz sands was fixed between two layers of quartz wool to maintain a 10 mm height of the bed (Supplementary Fig. 6c). The feed gases consisted of CH_4_ (hydrocarbons or CO), O_2_ and He were controlled individually by three mass flow controllers (Brooks, USA) to vary the total flows and partial pressures of the reactants. The product components leaving the reactor were monitored by an on-line gas chromatograph (Agilent 7890A, USA) with flame ionization detector and thermal conductivity detector. Blank experiments showed that reaction rates were negligible without catalyst.

## Additional information

**How to cite this article**: Wang, H. *et al.* *In situ* oxidation of carbon-encapsulated cobalt nanocapsules creates highly active cobalt oxide catalysts for hydrocarbon combustion. *Nat. Commun.* 6:7181 doi: 10.1038/ncomms8181 (2015).

## Supplementary Material

Supplementary Information Supplementary Figures 1-6, Supplementary Table 1 and Supplementary References
