# Supplementary material for: In situ oxidation of carbon-encapsulated cobalt nanocapsules creates highly active cobalt oxide catalysts for hydrocarbon combustion
Source: Nat Commun. 2015 Jun 15;6:7181. doi: 10.1038/ncomms8181 (PMC4490405; doi:10.1038/ncomms8181)
Supplement: Supplementary Information — Supplementary Figures 1-6, Supplementary Table 1 and Supplementary References [file ncomms8181-s1.pdf]

Supplementary Information:

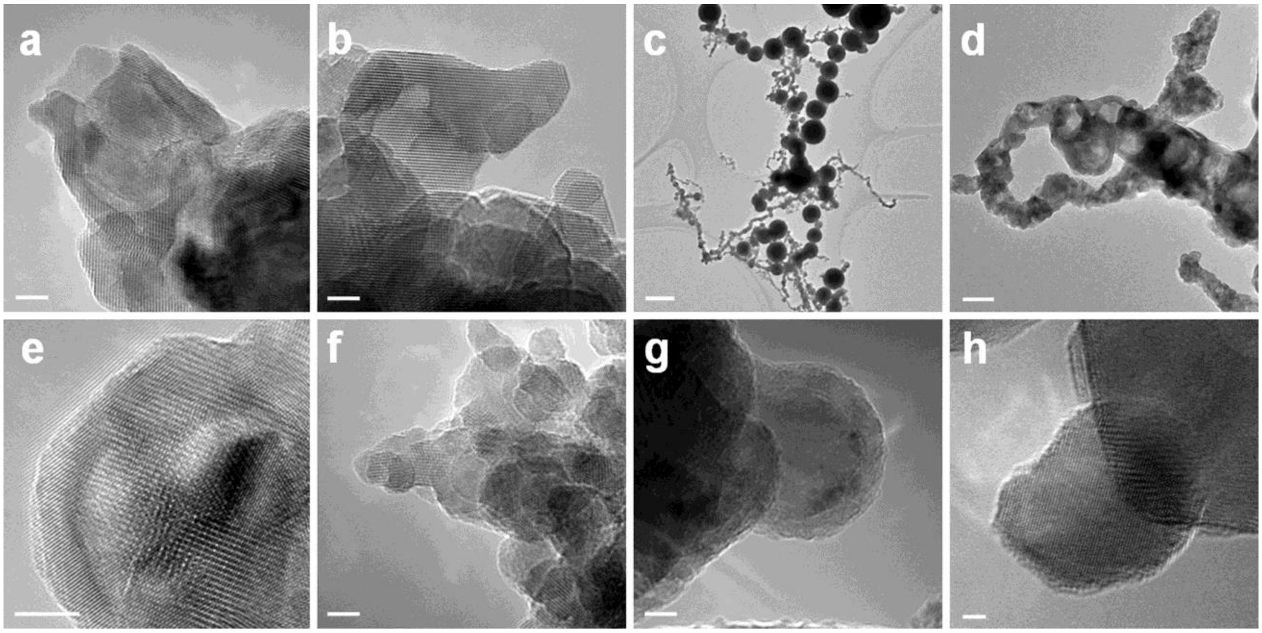

Supplementary Figure 1. TEM images. Co@C nanocapsules oxidized at (**a, e**) 300 ^{o}C and (**b, f**) 400 ^{o}C. Carbon-free Co nanoparticles oxidized at (**c, g**) 20 ^{o}C and (**d, h**) 450 ^{o}C. The carbon-free Co nanoparticles were synthesized by the same process without using ethanol. Scale bars, 5 nm (in **a, b, e-g**), 500 nm (in **c**), 50 nm (in **d**) and 2 nm (in **h**).

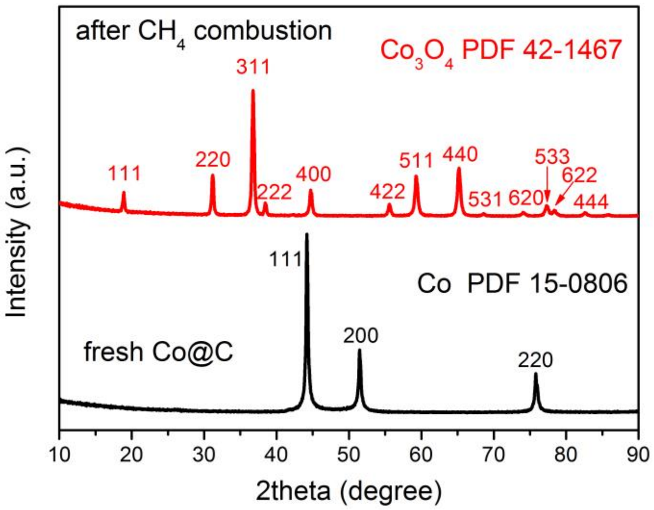

Supplementary Figure 2. Powder XRD patterns of the fresh and reacted Co@C samples. The curves of fresh and reacted samples were matched well with that of metallic cobalt (PDF 15-0806) and cobalt oxide (PDF 42-1467), respectively.

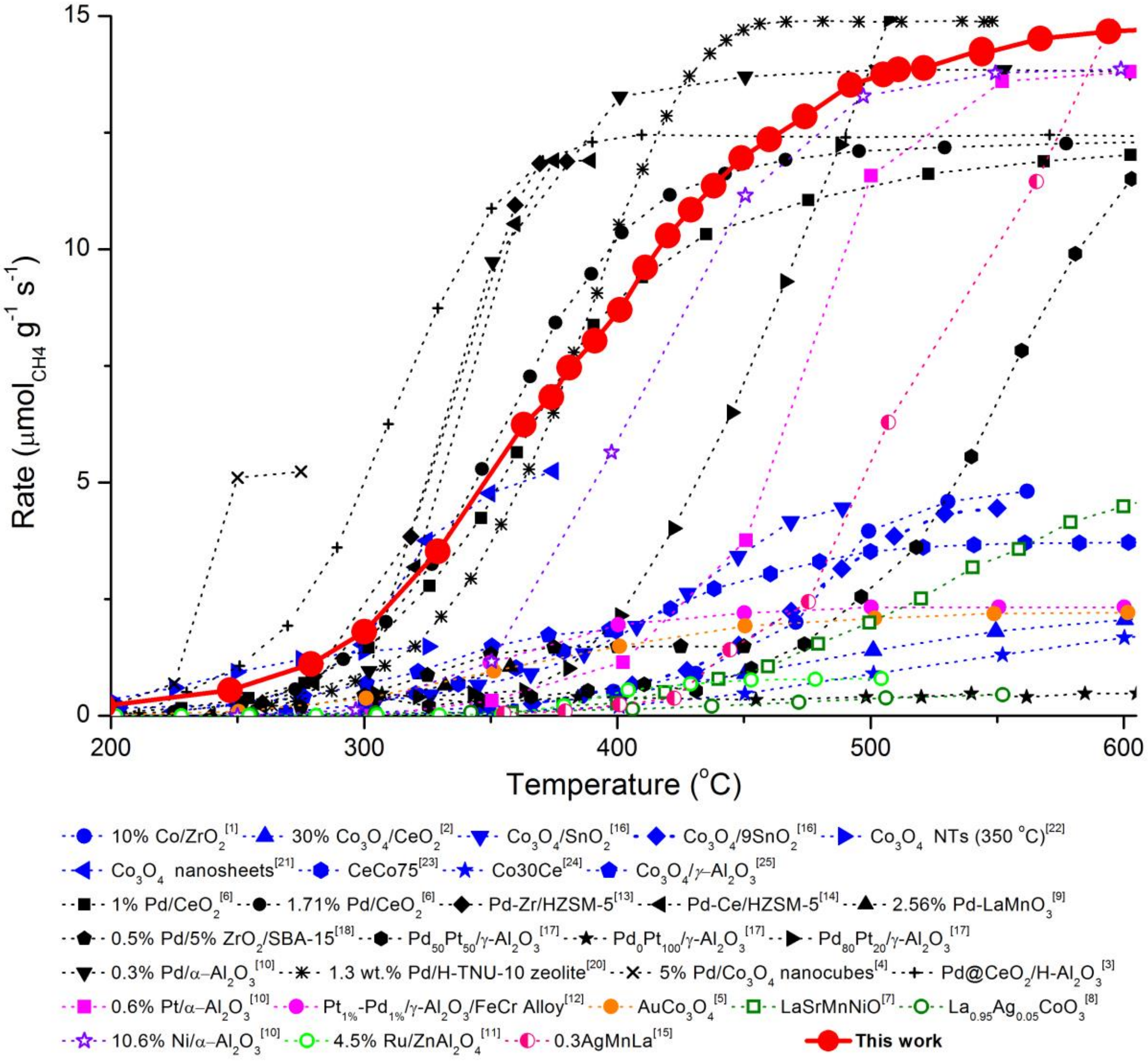

Supplementary Figure 3. Catalytic performance comparison with references[1-25]. The CH_4 reaction rates were calculated based on the total mass of these catalysts.

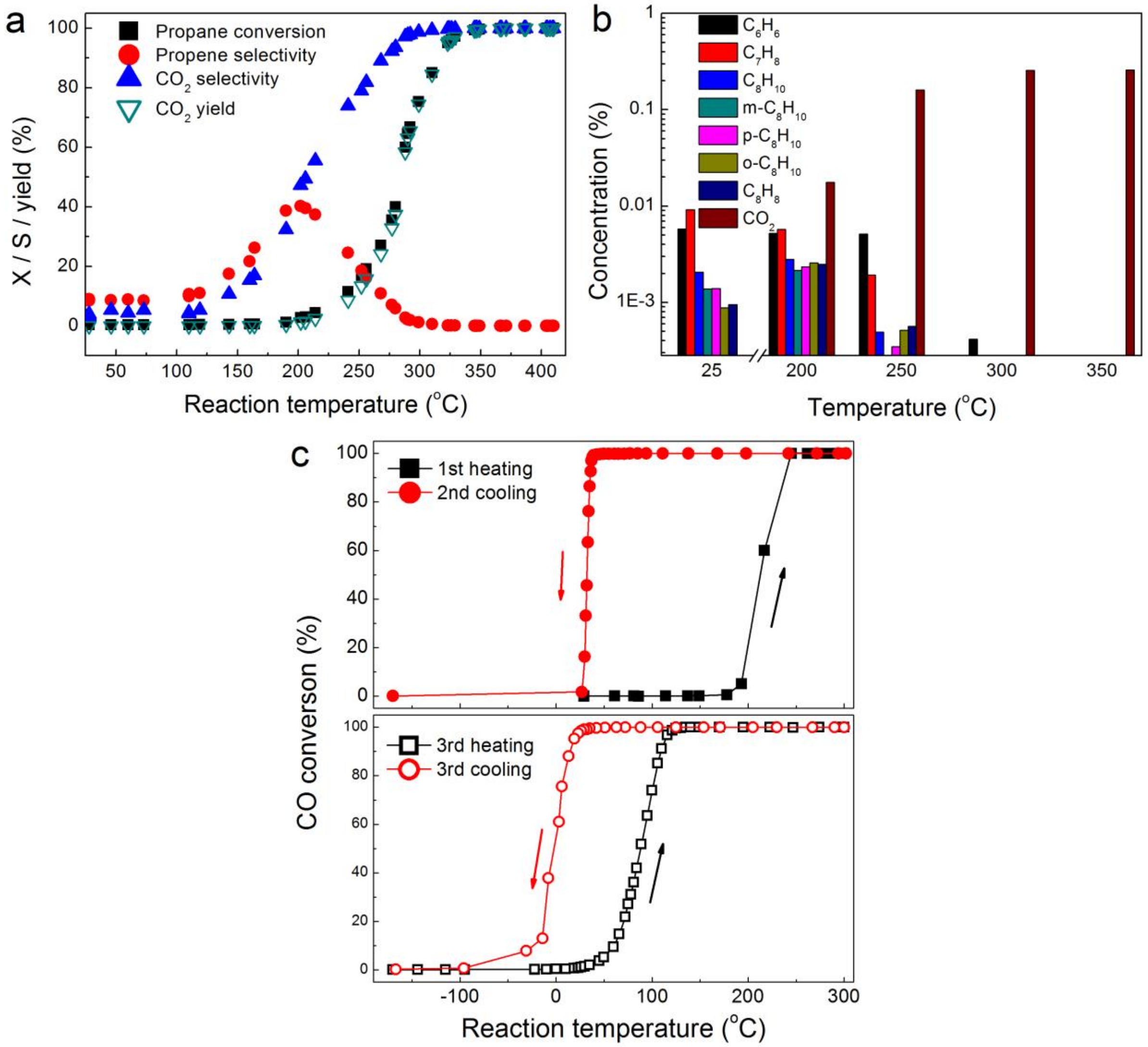

Supplementary Figure 4. Catalytic performances in combustion of propane, aromatics and carbon monoxide. (**a**) Propane conversion (*X*), propene selectivity (*S*), CO_2 selectivity (*S*) and yield as a function of reaction temperature obtained from room temperature to 420 °C over 100 mg Co@C catalyst, 2.6% C_3H_8 with excess oxygen. (**b**) Concentration of aromatic mixture and yielded CO_2 against the reaction temperature over 100 mg Co@C catalyst with excess oxygen. (**c**) Heating and cooling light-off curves of CO oxidation against the reaction temperature over 100 mg Co@C with 1.24% CO and 3.10% O_2 in helium at the total GHSV of 12.2 L g^{-1} h^{-1}. We note that the residual carbon shells may block the diffusion of reactant onto the active sites, resulting in an underestimation of the catalytic activity.

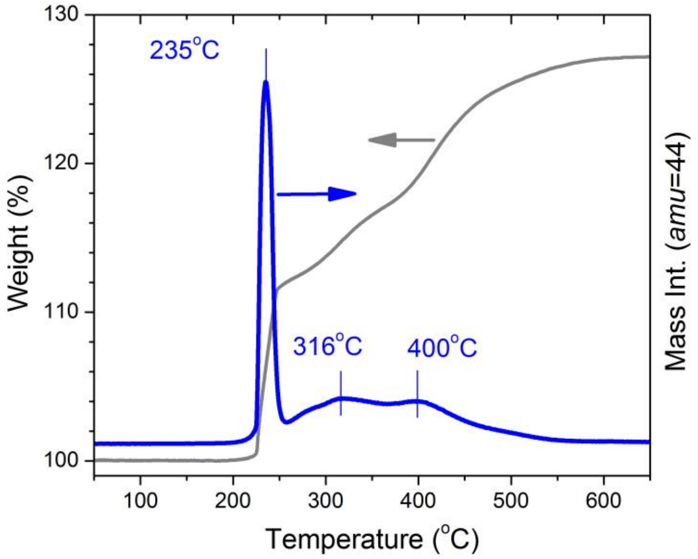

Supplementary Figure 5. Temperature-programmed oxidation of pristine Co@C sample in a flow of diluted oxygen. The pristine nanocapsules showed the chemical and thermal stability at the low temperature below 200 °C. When the temperature elevated from 220 °C to 600 °C, a total weight gain of about 27 wt% on the TG curve arises with synchronous changes in escaping CO_2 MS signals.

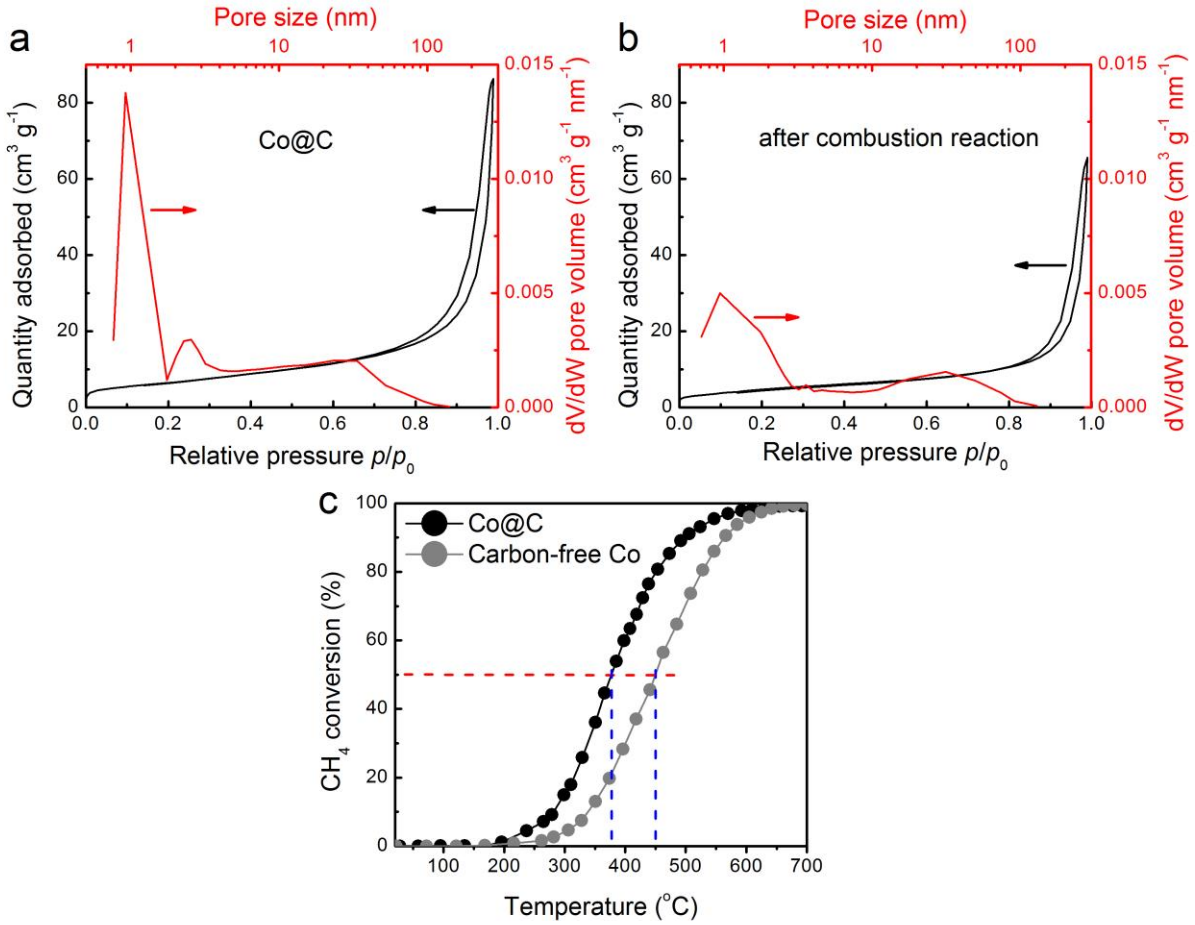

Supplementary Figure 6. Textural structure and catalytic performance of Co@C and carbon-free Co nanoparticles. N_2 physisorption results of Co@C samples (**a**) before and (**b**) after reaction. (**c**) Light-off curves of Co@C and carbon-free Co. We noted that the BET surface area and pore volume were 23.3 m^2 g^{-1}, 0.134 mL g^{-1} for the pristine Co@C and 17.5 m^2 g^{-1}, 0.102 mL g^{-1} for the used sample, respectively. The T_{50} values of catalytic combustion reaction were 376 ^{o}C and 450 ^{o}C, while the CH_4 conversion were 50% and 20.2% at 376 ^{o}C on Co@C and carbon-free Co catalysts, respectively.

Supplementary Table 1. Comparison of TOF and kinetic parameters with references.

**Catalyst**	**TOF** (s^{-1})	**CH_4 reaction order**	**O_2 reaction order**	**Activation energy** kJ mol^{-1}	**Reference**
Co_3O_4	0.016[a] (533K)	0.652	0.003	68 ± 1	This work
Co(1.9)/ZrO_2	—	—	—	108.68	26
Co(0.9)La/ZrO_2	—	—	—	121.22	
Co–Mg/Al ternary hydrotalcites	—	—	—	86~134	27
aCoO_3 (a=La, Pr, Nd, and Gd)	—	—	—	98.6~110	28
ZrLaMn_x (x=2,4,6,12,16)	—	0.79~0.82	—	85.2~100.9	29
LaMnO_3	—	0.83	—	97.5	
Pt/γ-Al_2O_3	0.0015	—	—	67~138	30
Pd wire	5.4 (673K)	0.8	0.1	71.0	31
Pt wire	0.13 (773K)	1.0	-0.6	87.8	
Rh wire	0.52 (773K)	0.6	0	100	
0.038~1.0wt% Pd/Al_2O_3	0.024~0.31 (673K)	0.6~0.8	0~0.1	71~84	
0.22wt% Pt/Al_2O_3	0.048~0.14 (773K)	1.1~1.2	-0.6 ~ -0.5	100	
0.03~1.153wt% Rh/Al_2O_3	0.048~0.14 (773K)	0.4~0.5	0~0.1	92~96	
Pd(111)	2.8-2.9 (598K)	0.8	-0.1	140 ± 20	32
Pd(100)	5.0-5.3 (598K)	0.8	0.1	125 ± 15	
Pd(110)	1.2-1.4 (598K)	0.7	0.2	160 ± 20	
2.7wt% Pd/γ-Al_2O_3	2.28 (623K)	1.0	0	84 ± 3 (<680 K); 23 ± 2 (>680 K)	33
3.3wt% Pd/γ-Al_2O_3	0.03-0.29 (473K)	0.95	0.86	78.6 ± 5.0	34
1.0~10.0wt% Pd/γ-Al_2O_3	—	1.0	0	76 (473-673K)	35
Pd/H-TNU-10	0.171 (673K)	0.6	0.0	78	20
Pd/H-ZSM-5(II)	0.109 (673K)	0.5	-0.2	84	
Pd/H-mordenite	0.075 (673K)	0.7	-0.1	77	
Pd/H-beta	0.068 (673K)	0.5	0.2	72	
Pd(1%)@CeO_2(9%)/H-Al_2O_3	0.047	—	—	103 (493~543K)	3
Pd(1%)/CeO_2 IWI	0.0013	—	—	90(493~543K)	
Pd(1%)/CeO_2(9%)/Al_2O_3 IMP	0.0015	—	—	120(523~563K)	
0.5~4.5wt%Ru/ZnAl_2O_4	0.006~0.010 (648K)	—	—	124~129	11

[a] The surface area of cobalt was measured by the CO chemisorption test according to Ref. 36. The density of active sites is 51.9 μmol_{Co} g^{-1}.

Supplementary References

1. Xiao, T. C., Ji, S. F., Wang, H. T., Coleman, K. S. & Green, M. L. H. Methane combustion over supported cobalt catalysts. *J. Mol. Catal. A* **175,** 111-123 (2001)
2. Liotta, L. F., Di Carlo, G., Pantaleo, G. & Deganello, G. Catalytic performance of Co_3O_4/CeO_2 and Co_3O_4/CeO_2-ZrO_2 composite oxides for methane combustion: Influence of catalyst pretreatment temperature and oxygen concentration in the reaction mixture. *Appl. Catal. B* **70**, 314-322 (2007)
3. Cargnello, M. *et al.* Exceptional activity for methane combustion over modular Pd@CeO_2 subunits on functionalized Al_2O_3. *Science* **337**, 713-717 (2012)
4. Hu, L. H., Peng, Q. & Li, Y. D. Low-temperature CH_4 catalytic combustion over Pd catalyst supported on Co_3O_4 nanocrystals with well-defined crystal planes. *Chemcatchem* **3**, 868-874 (2011)
5. Liotta, L. F., Di Carlo, G., Longo, A., Pantaleo, G. & Venezia, A. M. Support effect on the catalytic performance of Au/Co_3O_4-CeO_2 catalysts for CO and CH_4 oxidation. *Catal. Today* **139**, 174-179 (2008)
6. Colussi, S. *et al.* Nanofaceted Pd-O Sites in Pd-Ce surface superstructures: Enhanced activity in catalytic combustion of methane. *Angew. Chem. Int. Ed.* **48**, 8481-8484 (2009)
7. Alifanti, M., Kirchnerova, J., Delmon, B. & Klvana, D. Methane and propane combustion over lanthanum transition-metal perovskites: Role of oxygen mobility. *Appl. Catal. A* **262**, 167-176 (2004)
8. Buchneva, O. *et al.* La-Ag-Co perovskites for the catalytic flameless combustion of methane. *Appl. Catal. A* **370**, 24-33 (2009)
9. Cimino, S., Casaletto, M. P., Lisi, L. & Russo, G. Pd-$LaMnO_3$ as dual site catalysts for methane combustion. *Appl. Catal. A* **327**, 238-246 (2007)
10. Li, B. T. *et al.* High combustion activity of methane induced by reforming gas over Ni/Al_2O_3 catalysts. *Appl. Catal. A* **290**, 36-45 (2005)
11. Okal, J. & Zawadzki, M. Catalytic combustion of methane over ruthenium supported on zinc aluminate spinel. *Appl. Catal. A* **453**, 349-357 (2013)
12. Maione, A., Andre, F. & Ruiz, P. Structured bimetallic Pd-Pt/γ-Al_2O_3 catalysts on FeCrAlloy fibers for total combustion of methane. *Appl. Catal. B* **75**, 59-70 (2007)
13. Shi, C. K., Yang, L. F., He, X. E. & Cai, J. X. Enhanced activity and stability of Zr-promoted Pd/HZSM-5 catalyst for low-temperature methane combustion. *Chem. Commun.* 2006-2007 (2002)
14. Shi, C. K., Yang, L. F. & Cai, J. X. Cerium promoted Pd/HZSM-5 catalyst for methane combustion. *Fuel* **86**, 106-112 (2007)
15. Machocki, A. *et al.* Manganese-lanthanum oxides modified with silver for the catalytic combustion of methane. *J. Catal.* **227**, 282-296 (2004)
16. Tang, X. F., Hao, J. M. & Li, J. H. Complete oxidation of methane on Co_3O_4-SnO_2 catalysts. *Front. Environ. Sci. Engin. China* **3**, 265-270 (2009)
17. Persson, K., Ersson, A., Jansson, K., Fierro, J. L. G. & Jaras, S. G. Influence of molar ratio on Pd-Pt catalysts for methane combustion. *J. Catal.* **243**, 14-24 (2006)
18. Yin, F. X., Ji, S. F., Wu, P. Y., Zhao, F. Z. & Li, C. Y. Deactivation behavior of Pd-based SBA-15 mesoporous silica catalysts for the catalytic combustion of methane. *J. Catal.* **257**, 108-116 (2008)
19. Rui, Z. B., Huang, Y. F., Zheng, Y., Ji, H. B. & Yu, X. Effect of titania polymorph on the properties of CuO/TiO_2 catalysts for trace methane combustion. *J. Mol. Catal. A* **372**, 128-136 (2013)
20. Park, J. H. *et al.* Methane combustion over Pd catalysts loaded on medium and large pore zeolites. *Top. Catal.* **52**, 27-34 (2009)

21. Hu, L. H., Peng, Q. & Li, Y. D. Selective synthesis of Co_3O_4 nanocrystal with different shape and crystal plane effect on catalytic property for methane combustion. *J. Am. Chem. Soc.* **130**, 16136-16137 (2008)

22. Fei, Z. Y., He, S. C., Li, L., Ji, W. J. & Au, C. T. Morphology-directed synthesis of Co_3O_4 nanotubes based on modified Kirkendall effect and its application in CH_4 combustion. *Chem. Commun.* **48**, 853-855 (2012)

23. Li, H. F. *et al.* Catalytic methane combustion over Co_3O_4/CeO_2 composite oxides prepared by modified citrate Sol-Gel method. *Catal. Lett.* **141**, 452-458 (2011)

24. Liotta, L. F., Di Carlo, G., Pantaleo, G., Venezia, A. M. & Deganello, G. Co_3O_4/CeO_2 composite oxides for methane emissions abatement: Relationship between Co_3O_4-CeO_2 interaction and catalytic activity. *Appl. Catal. B* **66**, 217-227 (2006)

25. Zavyalova, U., Scholz, P. & Ondruschka, B. Influence of cobalt precursor and fuels on the performance of combustion synthesized Co_3O_4/γ-Al_2O_3 catalysts for total oxidation of methane. *Appl. Catal. A* **323**, 226-233 (2007)

26. Iamarino, M., Chirone, R., Pirone, R., Russo, G. & Salatino, P. Catalytic combustion of methane in a fluidized bed reactor under fuel-lean conditions. *Combust. Sci. Technol.* **174**, 361-375 (2002)

27. Jiang, Z. *et al.* Catalytic combustion of methane over mixed oxides derived from Co-Mg/Al ternary hydrotalcites. *Fuel Process. Technol.* **91**, 97-102 (2010)

28. Baiker, A., Marti, P. E., Keusch, P., Fritsch, E. & Reller, A. Influence of the a-Site Cation in $ACoO_3$ (a=La, Pr, Nd, and Gd) Perovskite-Type Oxides on Catalytic Activity for Methane Combustion. *J. Catal.* **146**, 268-276 (1994)

29. Cimino, S. *et al.* Methane combustion and CO oxidation on zirconia-supported La, Mn oxides and $LaMnO_3$ perovskite. *J. Catal.* **205**, 309-317 (2002)

30. Beck, I. E. *et al.* Platinum nanoparticles on Al_2O_3: Correlation between the particle size and activity in total methane oxidation. *J. Catal.* **268**, 60-67 (2009)

31. Yao, Y. F. Y. Oxidation of Alkanes over Noble-Metal Catalysts. *Ind. Eng. Chem. Prod. Res. Dev.* **19**, 293-298 (1980)

32. Zhu, G. H., Han, J. Y., Zemlyanov, D. Y. & Ribeiro, F. H. The turnover rate for the catalytic combustion of methane over palladium is not sensitive to the structure of the catalyst. *J. Am. Chem. Soc.* **126**, 9896-9897 (2004)

33. Cullis, C. F. & Willatt, B. M. Oxidation of Methane over Supported Precious Metal-Catalysts. *J. Catal.* **83**, 267-285 (1983)

34. Xu, J. *et al.* Operando and Kinetic Study of Low-Temperature, Lean-Burn Methane Combustion over a Pd/γ-Al_2O_3 Catalyst. *ACS Catal.* **2**, 261-269 (2012)

35. Ribeiro, F. H., Chow, M. & Dallabetta, R. A. Kinetics of the Complete Oxidation of Methane over Supported Palladium Catalysts. *J. Catal.* **146**, 537-544 (1994)

36. Zhu, Z. Z. et al. Highly active and stable Co_3O_4/ZSM-5 catalyst for propane oxidation: effect of the preparation method. *ACS Catal.* **3**, 1154-1164 (2013)
